# Augmentation of Reverse Transcription by Integrase through an Interaction with Host Factor, SIP1/Gemin2 Is Critical for HIV-1 Infection

**DOI:** 10.1371/journal.pone.0007825

**Published:** 2009-11-13

**Authors:** Hironori Nishitsuji, Takaya Hayashi, Takuya Takahashi, Masashi Miyano, Mari Kannagi, Takao Masuda

**Affiliations:** Department of Immunotherapeutics, Graduate School of Medicine and Dentistry, Tokyo Medical and Dental University, Tokyo, Japan; Yonsei University, Republic of Korea

## Abstract

There has been accumulating evidence for the involvement of retroviral integrase (IN) in the reverse transcription of viral RNA. We previously identified a host factor, survival motor neuron-interacting protein 1 (SIP1/Gemin2) that binds to human immunodeficiency virus type 1 (HIV-1) IN and supports HIV-1 infection apparently at reverse transcription step. Here, we demonstrated that HIV-1 IN together with SIP1 augments reverse transcriptase (RT) activity by enhancing the assembly of RT on viral RNA *in vitro*. Synthetic peptides corresponding to the binding motifs within IN that inhibited the IN-SIP1 interaction abrogated reverse transcription *in vitro* and *in vivo*. Furthermore, knockdown of SIP1 reduced intracellular stability and multimer formation of IN through proteasome-mediated degradation machinery. Taken together, SIP1 appears to stabilize functional multimer forms of IN, thereby promoting the assembly of IN and RT on viral RNA to allow efficient reverse transcription, which is a prerequisite for efficient HIV-1 infection.

## Introduction

Upon infection of host cells with a retrovirus, the viral genome is subjected to several processes that include uncoating, reverse transcription of the viral genomic RNA into a cDNA copy by reverse transcriptase (RT), transport of this cDNA into the nucleus, and integration of the cDNA into the host chromosome. These early events are mediated through the interactions of several viral proteins and host factors with the viral genome, often referred as reverse transcription complex (RTC) or preintegration complex (PIC) [1], [2]. The cDNA copy of the viral genome integrates into a host cell chromosome by integrase (IN) [3]. Several cellular proteins have been reported to interact directly with HIV-1 IN, including integrase interactor 1 [4], lens epithelium-derived growth factor/transcription co-activator p75 (LEDGF/p75) [5] for chromosomal targeting of HIV-1 IN [6]–[8]. More recently, von Hippel-Lindau binding protein 1 (VBP1), a subunit of the prefoldin chaperone, has been identified as an IN cellular binding protein that bridges interaction between IN and the cullin2 (Cul2)-based von Hippel-Lindau (VHL) ubiquitin ligase [9]. VBP1 and the Cul2/VHL ligase cooperate in the efficient polyubiquitylation of IN and its subsequent proteasome-mediated degradation that is perquisite for efficient transcription from integrated viral DNA.

Putative roles for IN at steps prior to integration, such as uncoating [10]–[12], reverse transcription [11], [13]–[15], and nuclear import of viral cDNA [14], [16], [17] have been suggested. Although the mechanisms for these pleiotropic effects of IN mutations are largely unknown, there has been accumulating evidence for the involvement of retroviral INs in the reverse transcription [11], [13], [14], [18], [19]. Contribution of IN during the reverse transcription has also been noticed in a retrovirus like element of *Saccharomyces cerevisiae*, Ty3 [20], [21]. A previous study from our laboratory showed that reverse transcription of HIV-1 was abrogated by knocking down a host factor, survival motor neuron (SMN)-interacting protein 1 (SIP1/Gemin2) that binds to HIV-1 IN [22]. SIP1/Gemin 2 is a component of the SMN complex that mediates the assembly of spliceosomal small nuclear ribonucleoproteins (snRNPs) and nucleolar ribonucleoproteins (snoRNP) [23]–[27].

In the present study, we identified critical residues within IN for interaction with SIP1. Interruption of the IN-SIP1 interaction through introducing the mutations in HIV-1 IN or with synthetic peptides corresponding to the binding motif of the IN for SIP1 resulted in abrogation of reverse transcription, indicating that IN-SIP1 interaction is a prerequisite for efficient HIV-1 infection. For mechanistic insight of the SIP1-IN interaction, we demonstrate that HIV-1 IN and SIP1 synergistically stimulate RT activity by enhancing the assembly of RT on viral RNA *in vitro*. Furthermore, we also showed that SIP1 stabilizes the formation of a functional multimer of IN, thereby promoting the assembly of IN and RT on viral RNA to allow an efficient reverse transcription. Our findings will shed light on the mechanism of the functional role of IN during reverse transcription of the retroviral genome and could serve as a basis for a novel therapeutic approach to treat HIV-1 disease.

## Results

### Intracellular Interaction between HIV-1 IN and SIP1

**Figure 1:**
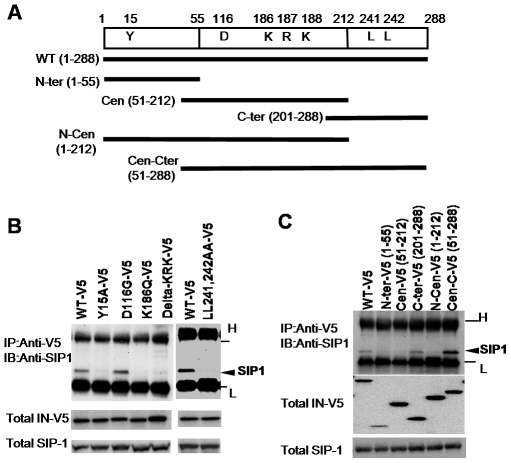
Intracellular interaction between SIP1 and IN mutants resulting in a reverse transcription defective phenotype. (A) Schematic diagram of HIV-1 integrase. Numbers refer to amino-acid residues of IN. The locations of the critical amino acids for reverse transcription *in vivo* (Tyr15, Lys 186, Arg187 and Lys188, and LeuLeu241,242) and the active site residues Asp116 are indicated. The region of each truncated form of IN is shown as a solid line. (B, C) Wild type (WT) or IN mutant expression plasmids with a V5-tag were transfected into 293T cells. For transfection, 1.0 µg of each plasmid for WT-V5, D116G, N-ter, Cen, C-ter, N-Cen or Cen-C, and 2.5 µg for Y15A, K186Q, delta-KRK or LL241,242AA was used, respectively. At 48 h after transfection, cells were harvested and lysed with RSB-100 containing 1.0% NP-40. Cell lysate was then subjected to immunoprecipitation using an anti-V5 antibody, followed by Western blot analysis with an anti-SIP1 antibody. H and L denote heavy and light chains of immunoglobulin, respectively.

Previous studies showed that mutations in the conserved amino acid residues of HIV-1 IN abolished reverse transcription of viral genomic RNA after infection [11], [12], [14], [18]. Firstly, we examined the effect of these IN mutations including Y15A [28], K186Q, Delta KRK [14], and LL241,242AA [18] on their intracellular interaction with endogenous SIP1 (Figure 1A). For this assay, an IN protein with the V5 epitope at its COOH-terminus was expressed in 293T cells, then subjected to immunoprecipitation with an anti-V5 antibody followed by Western blotting using an anti-SIP1 antibody. A specific interaction of the wild type IN-V5 (WT-V5) with SIP1 was detected (Figure 1B). When variants of IN-V5 carrying each mutation were expressed in 293T cells, levels of the Y15A-V5, K186Q-V5 and LL241,242AA-V5 IN variants were significantly lower than that of WT (Figure S1A). Therefore, the amount of mutant plasmid vector used was increased to result in steady-state levels of the IN-V5 variant proteins similar to that of the WT-V5 control. Under these experimental conditions, all of the IN carrying mutation of Y15A, K186Q, Delta-KRK or LL241,242AA significantly reduced binding to endogenous SIP1 (Figure 1B). Interestingly, we noticed that deletion of the KRK 186–188 residues (Delta-KRK) increased the steady-state level of IN significantly (Figure S1A) and that introduction of the Delta-KRK mutation in Y15A mutant (Y15A-delKRK) restored the stability (Figure S1B). These results suggest that the KRK residues might be critical for proteasome-mediated degradation that accompanied with loss of SIP1 interaction as shown in the following experiments. In parallel, IN-V5 carrying a D116G mutation at the IN catalytic sites was also tested, since HIV-1 with a D116G variant of IN is defective for proviral integration but retains normal level of cDNA synthesis (reverse transcription) activity [11], [29], [30]. D116G-V5 interacted with endogenous SIP1 as efficiently as WT-V5. Thus, all of the IN mutations that cause reverse transcription defective phenotype, at least tested here, significantly reduced the intracellular interaction with SIP1, suggesting that the interaction of IN with SIP1 is critical for efficient reverse transcription in the HIV-1 infection cycle. Next, to map the domain(s) of IN that are essential for interaction with SIP1, a series of truncated forms of IN were examined. Consistent with our previous pull-down study using recombinant GST-IN fusion protein [22], the COOH-terminal domain of IN (IN-Cter, 201–288) and full-length IN (IN1–288) each bound to SIP1 efficiently (Figure 1C). The NH2-terminal domain of IN (IN1–55) and the central core domain of IN (IN51–212) showed much weaker affinity compared to the C-terminal domain (IN201–288). Of note, the addition of the central core domain to the C-terminal domain (IN51–288) significantly enhanced binding with SIP1. These results suggest that both the central core and the C-terminal domains of HIV-1 IN contribute to efficient binding to SIP1 within cells.

### Direct Interaction between Recombinant IN and SIP1 Proteins *In Vitro*

**Figure 2:**
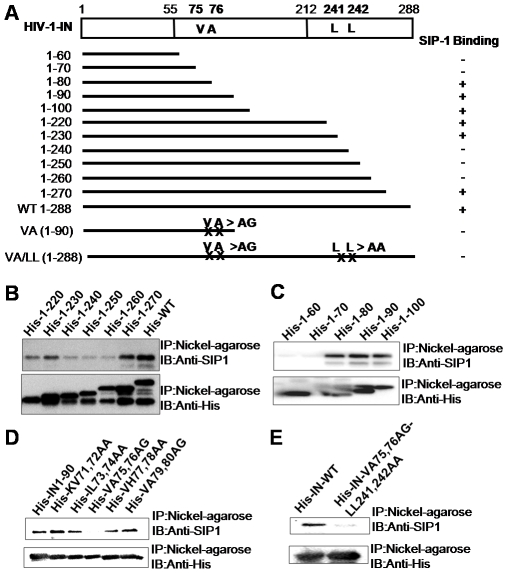
Residues of HIV-1 IN that are critical for specific interactions with recombinant SIP1 *in vitro*. (A) A schematic of the full length (open bar, top) of HIV-1 IN and its deletion forms (solid line) are shown. Point mutations of the residues of IN (Val75, Ala76, Leu241 and Leu242) that are critical for interaction with SIP1, are indicated with their location numbers. (B, C) For pull-down experiments, 10 µg of recombinant SIP1 was incubated with 10 µg of each His-tagged IN (His-IN), followed by purification through a nickel agarose column. The level of SIP1 bound to each IN-nickel agarose complex was determined by Western blot analysis using an anti-SIP1 antibody. (D) Series of amino acid substitutions targeting the residues from 71 to 80 of the His-tagged IN (1–90) protein were generated. Interaction of each mutant protein with SIP1 was determined as described above in (B). (E) Full-length His-tagged IN mutant proteins carrying the VA74,75AG or LL241,242AA mutations were generated. Interaction of each mutant protein with SIP1 was determined as described above in (B).

We then evaluated the direct interaction between HIV-1 IN and SIP1 by using recombinant IN and SIP1 proteins *in vitro*. Recombinant His-tagged IN protein with full size (His-WT) or a series of deletions (Figure 2A) was incubated with recombinant SIP1 (rSIP1). The reaction mixture was then subjected to nickel-column purification followed by Western blotting using an anti-SIP1 antibody. The His-WT and His-1–270 variant in which the 18 amino acids from the C-terminal of IN were deleted showed efficient interaction with rSIP1 *in vitro*. Further deletions of the C-terminus (His-1–260, -1–250, and -1–240) significantly reduced binding to rSIP1. This finding suggests that the residues between 240 to 260 within the C-terminal domain of IN are critical for the direct interaction with SIP1. Curiously, further deletion of residues from the C-terminus of His-IN (His-1–230, 1–220) significantly restored binding of IN to rSIP1 (Figure 2B), suggesting that there is an additional binding motif outside of the C-terminal domain of HIV-1 IN. It is also plausible that in the absence of the C-terminal residues, the residues 240–260 may be inhibitory to the binding motif outside of the C-terminal domain of HIV-1 IN. However, as demonstrated in the following experiments, the peptide corresponding to the region (IN231–251) inhibit the IN-SIP1 interaction and its RT-stimulatory effects, indicating contribution of the residues of 240–260 for functionally relevant binding to SIP1 at least in the full length form of HIV-1 IN. On the other hand, residues of 1–60 (His-1–60) or 1–70 (His-1–70) did not bind to rSIP1, while residues of His-1–80, -1–90, and -1–100 exhibited significant binding to rSIP1 (Figure 2C). Thus, residues of 71–80 in the central core domain of HIV-1 IN are also critical for the interaction with SIP1.

Further mutational analyses targeting the residues of 71–80 revealed that Val75 and Ala76 were critical for the interaction of the IN1–90 form with rSIP1 (Figure 2D). However, in the context of full-length form of IN, mutations of the Val75-Al76 residues (VA75,76AG) did not affect the binding of full-length IN to rSIP1. Similarly, none of the Y15A, K186Q, or LL241,242AA mutations that affected the intracellular interaction of HIV-1 IN and SIP1 (Figure 1B) affected the interaction *in vitro* (Figure S2). However, the full-length IN protein carrying both of the VA75,76AG and LL241,242AA mutations exhibited significantly reduced binding to rSIP1 *in vitro* (Figure 2E). These results show that the Val75-Ala76 and Leu241-L242 residues of HIV-1 IN are critical for direct binding with SIP1. The apparent discrepancy between the *in vivo* and *in vitro* binding assays could be due to different folding of the protein under the two different conditions, suggesting that the Y15A, K186Q, or LL241–242AA mutations might affect the conformation or the higher-order structure of HIV-1 IN that is required for efficient interaction with SIP1 *in vivo*. It is also possible that additional motifs including the residues of Y15, K186 and LL241–242 and/or factor(s) might be involved for the IN-SIP1 interaction *in vivo*.

### Characterization of Intracellular IN Proteins in SIP1 Knock-Down Cells

**Figure 3:**
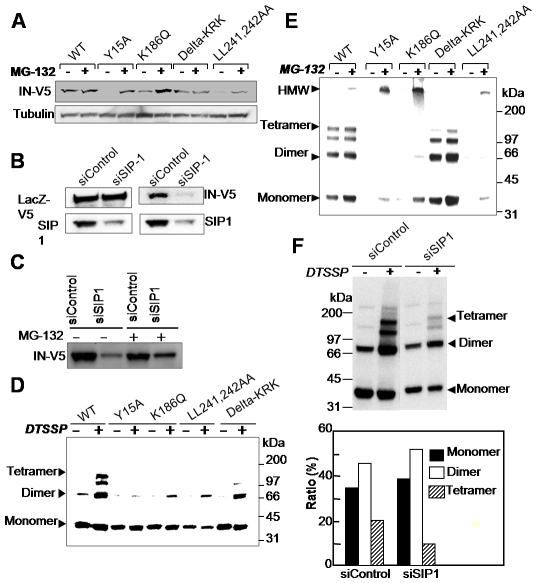
Characterization of intracellular IN proteins. (A) 293T cells were transfected with indicated plasmid in presence of 10 µM MG-132 (+) or DMSO(−). Total cell lysates (10 µg) were analyzed by Western blotting analysis. (B, C) 293T cells were transfected twice with 100 pmol of siControl or siSIP1 and then with lacZ-V5 or an IN-V5 expression plasmid with MG-132 or DMSO. 48 h later, cells were lysed with CSK buffer containing 0.5% NP-40 followed by Western blotting analysis. (D, E) 293T cells transfected each IN-V5 expression vector were suspended with CSK buffer containing 0.5% NP-40. Cell lysates were incubated in either the absence or presence of 0.2 mM DTSSP at room temperature for 20 min and analyzed by Western blotting using an anti-V5 antibody. (F) Cell lysates in (B) were treated with 0.2 mM of DTSSP and then analyzed by Western blotting. The relative ratio of monomers, dimers, and tetramers after treatment with 0.2 mM DTSSP was analyzed using Image-J software (bottom). The relative ratio (%) of each form to the total level of IN-V5 is indicated.

Several groups have reported that IN is subject to degradation by the cellular ubiquitin-proteasome system [9], [31]. Thus, we examined whether SIP1 might act to protect HIV-1 IN from proteasomal degradation. In 293T cells, expression levels of mutants Y15A-V5, K186Q-V5 and LL241,242AA-V5 were lower than those of WT-V5 (Figure S1). Of note, treatment with the proteasome inhibitor MG132 restored the expression levels of the IN mutants (Figure 3A), suggesting that these mutants might be susceptible to proteasome-mediated degradation. We tested this hypothesis by using RNAi to specifically knockdown SIP1 in cells. The siRNA against SIP1 (siSIP1) significantly reduced the level of IN-V5 (Figure 3B). Under the same conditions, siSIP1 did not affect the level of LacZ-V5, an unrelated control protein. Of note, the level of IN-V5 in siSIP1-treated cells was restored by addition of MG132 (Figure 3C), suggesting that SIP1 might contribute to intracellular IN stability by protecting it from proteasome-mediated protein degradation. Interestingly, the expression level of the Delta-KRK-V5 mutant was high in both the presence and absence of MG132 treatment (Figure S1 and Figure 3A). Thus, the Delta-KRK mutation might affect the susceptibility of IN to ubiquitination, or other steps required for proteasome-mediated protein degradation. On the other hand, knocking down SIP1 did not affect the overall intracellular localization of IN-V5 (Figure S3). Importantly, the level of WT-V5 was not changed by treatment with MG132, suggesting that WT-V5 can form stable conformations that protect it from proteasome-mediated protein degradation through an interaction with SIP1 (Figure 3A). Of note, the Y15A and K186Q mutations were localized predominantly in cytoplasm (data not shown) and exhibited a low level of expression compared with WT-IN (Figure S1). Therefore, we hypothesize that SIP1 might exert its effect in the cytoplasm to stabilize functional IN conformations that are required for efficient viral cDNA synthesis.

It has been reported that HIV-1 IN functions as a dimer or other higher-order structure [32]–[35]. Cross-linking experiments using 3,3′-Dithiobis sulfosuccinimidylpropionate (DTSSP) revealed that the IN dimer, trimer or tetramer forms could be easily detected when wild type IN-V5 was expressed in 293T cells (Figure 3D). Meanwhile, Y15A-V5, K186Q-V5, or LL241–242AA IN mutants produced very low levels of the dimer or multimer forms under the same experimental conditions. Interestingly, the Delta-KRK mutant was detected as a dimer, but to a lesser extent as a tetramer. High molecular weight bands for the Y15A-V5, K186Q-V5, or LL241–242AA IN mutants were evident when the cross-linking experiment was performed in the presence of MG132 (Figure 3E). Since high molecular weight bands for the WT-V5 and Delta-KRK mutants were less evident in the presence of MG132, the high molecular weight bands might represent aggregated forms of IN proteins resulting from improper multimerization, which would be subject to proteasome-mediated degradation. These results suggest that proper multimerization, most probably in a tetramer formation of IN, might be required for efficient interaction with SIP1. Importantly, in SIP1 knock-down cells, IN-V5 dimer formation was still observed, although the total level of IN-V5 was reduced (Figure 3F, top). Of note, tetramer formation of IN-V5 was reduced to about 50% of the level in control siRNA treated cells (Figure 3F, bottom). Thus, SIP1 might contribute to the formation and/or stability of higher-ordered forms of HIV-1 IN, thereby protecting IN from proteasome-mediated protein degradation machinery.

### Augmentation of Reverse Transcription by HIV-1 IN and SIP1 *In Vitro*

**Figure 4:**
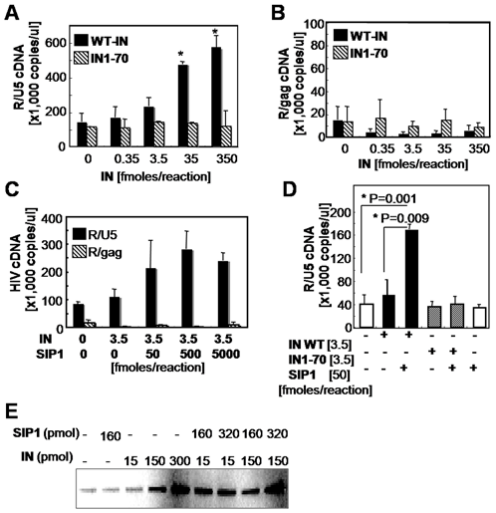
Augmentation of reverse transcription by HIV-1 IN and SIP1 *in vitro*. (A, B) The *in vitro* RT assay was carried out with 35 fmoles of HIV-1 RT (GST-RTp66) in the absence or presence of different amounts of full-length rIN (WT-IN). The truncated form of IN (IN1–70) that lack SIP1 binding domains was also tested in apparel as a control. The amount of HIV-1 cDNA was measured by real-time PCR using the HIV-1 R/U5 (A) or R/gag (B) primer pair [11]. Significant augmentation (p<0.05) by WT-IN in the levels of HIV-1 cDNA compared with the level without IN (0 fmole) was indicated by asterisks. (C) The in vitro RT assay was carried out as described in (A) in either the absence or presence of 3.5 moles of WT-IN, together with different amounts of rSIP1. (D) The in vitro RT assay was carried out with 3.5 fmoles of WT-IN (black bar) or IN1–70 (slash bar), in either the absence (−) or presence (+) of 50 fmoles of rSIP1. (E) The oligo(dT) cellulose resin was incubated with *in vitro* transcribed HIV-1 RNA for 1 h. After incubation, 10 µg of GST-RT in either the absence or presence of His-IN or rSIP1 was added and incubated for 2 h. After four washes with the IN storage buffer, precipitates were analyzed by immunoblot analysis using an anti-GST antibody.

Recently, Chow and colleagues have reported that HIV-1 IN plays an important role during the reverse-transcription step of the viral life cycle through physical interactions with RT [36]. Moreover, they have indicated that IN augments the initiation and elongation steps of HIV-1 reverse transcription *in vitro* [19]. We examined the functional role of HIV-1 IN and SIP1 during the reverse transcription of viral RNA using an *in vitro* cell-free RT assay. In this RT assay, *in vitro* transcribed RNA with a 5′m^7^ G Cap analog and 3′poly (A) tail, mimicking the HIV-1 genomic RNA in a virus particle, was used as a template RNA. For a primer, synthetic ribonucleotides that were designed to be annealed with the primer binding site (PBS) of HIV-1 were used. The amount of recombinant RT (35 fmoles per reaction) and the template RNA (0.04∼0.4 fmoles/reaction) was optimized to detect IN-mediated augmentation of RT activity. The level of the cDNA products (R/U5) was significantly increased by WT-IN at the dose of 35 (P = 0.02) and 350 fmoles per reaction (P = 0.03). Truncated form of HIV-1 IN (IN1–70) that lacks the central and C-terminal domains containing SIP1 binding regions, however, exhibited no significant stimulatory effect at any dose (P = 0.58∼0.98) (Figure 4A). Importantly, stimulation of RT activity by IN was further enhanced by SIP1 in a dose-dependent manner (Figure 4C). Significant stimulation of RT activity by SIP1 (P = 0.001) was evident when the full-length IN (IN-WT) was present (Figure 4D). However, SIP1 alone, nor with IN1–70 exhibited no significant (P = 0.69 and 0.98, respectively) stimulatory effect (Figure 4D and Figure S4A). The effect of SIP1 was not evident when higher amounts of the IN protein were added to the RT assay (Figure S4B), suggesting that SIP1 functions in IN-dependent manner. Thus, SIP1 might be required for the efficient recruitment of IN into the reverse transcription complex in the natural course of viral infection where the amounts of IN and RT are limiting.

To examine the sizes of the cDNA products in our RT assay, we carried out the in vitro RT assay with [α^32^P] dCTP and subjected to PAGE analysis. We obtained smear bands ranging from 50 to 180 nt, the amount of which was augmented by the IN and SIP1 (Figure S4C). Thus, most of the cDNA products in our RT assay might be the early species of cDNA including the minus strand strong-stop cDNA, expected size of which is ∼170 nt. Meanwhile, the level of the late cDNA products (R/gag) was always less than 1% of the total cDNA (R/U5) level at any dose of IN (Figure 4B). The R/gag cDNA products were detected even without PBS primer if high amount of RNA or RNA without heat-denature were used (data not shown). Therefore, most of the R/gag cDNA products might be synthesized by self-priming through an inter- or intra-hybridization of RNA templates by themselves. Of note, IN and SIP1 decreased the level of the R/gag products compared to IN1–70 control, suggesting that IN and SIP1 might increase the correct initiation of cDNA synthesis from the PBS primer. Taken together, stimulatory effect of IN and SIP1 on both of initiation and elongation of viral cDNA during the strong-stop cDNA synthesis.

Next, the effects of IN and SIP1 on the assembly of RT on viral RNA was examined. Briefly, *in vitro* transcribed HIV-1 RNA immobilized on oligo-d(T) cellulose resin was incubated with recombinant RT in either the presence or absence of IN and SIP1. Levels of RT on viral RNA were determined by immunoblot assay. In the presence of IN, the level of RT on viral RNA was increased in a dose-dependent manner. SIP1, together with IN, enhanced RT assembly on viral RNA (Figure 4E). Thus, HIV-1 IN stimulates RT activity together with SIP1 by enhancing or stabilizing RT assembly on viral RNA.

### Interruption of the SIP1-IN Interaction with Synthetic Peptides Corresponding to the Binding Motif of IN for SIP1

**Figure 5:**
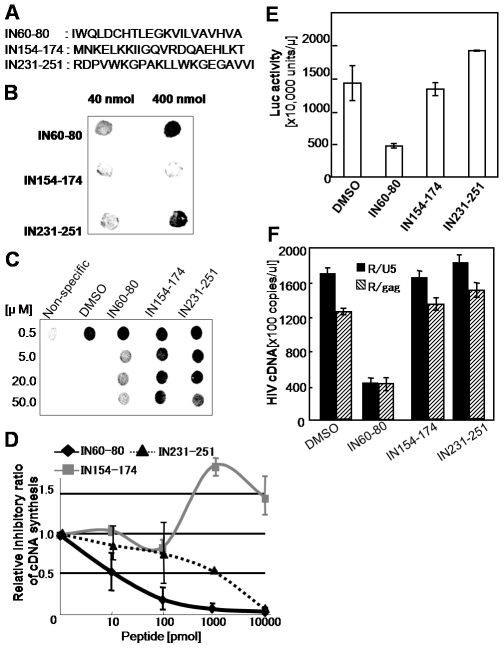
IN-derived peptide inhibits reverse transcription *in vitro* and *in vivo*. (A) Amino acid sequences of IN-derived peptides are indicated. (B) 40 or 400 nmol of each peptide bound to nitrocellulose filter was incubated with rSIP1 (5 µg). The rSIP1 bound to the peptide was detected using an anti-SIP1 antibody. (C) His-IN (10 µg) was bound to nitrocellulose and reacted with 5 µg of rSIP1 in the presence of each peptide at the indicated concentration. The rSIP1 bound to the peptide was detected as described in (B). (D) The *in vitro* reverse transcription assay was performed with 35 fmol of RT, 3.5 fmol of His-IN, and 50 fmol of rSIP1 in either the absence (control) or presence of IN-derived peptide at the indicated concentration. The values (mean±SE) plotted are the levels of HIV-1 cDNA relative to that the control, taken as 1.0. (E, F) PMA-stimulated THP-1 cells were treated with 100 µM of IN-derived peptide for 16 h. Cells were infected with HIV_NL43-luc_/VSV-G pseudotypes [11] in the presence of 100 µM of IN-derived peptide for 6 h. At 24 h post-infection, levels of viral gene expression in cells were determined by measuring the luciferase activity (E) and the levels of viral cDNA synthesis for early (R/U5, black bar) and late (R/gag, slash bar) products (F) were determined as described in Figure 4.

Binding assays revealed that residues in the central and the C-terminal domains of HIV-1 IN are critical for efficient binding to SIP1 (Figure 2). Synthetic peptides corresponding to the residues of 60–80 (IN60–80) and the residues of 231–251(IN 231–251) of HIV-1 IN were generated (Figure 5A). The direct binding of each peptide to SIP1 was evaluated using the dot-blot binding assay as described elsewhere [37]. Peptides corresponding to the residues of 154–174 (IN154–174), which contain a motif critical for the interaction with LEDGF/p75 [38], [39], were used as a control. Both peptides, IN60–80 and IN 231–251, efficiently bound to rSIP1 under conditions in which the control peptide, IN154–174, did not (Figure 5B). Furthermore, both of the IN60–80 and IN231–251 peptides significantly interfered with the binding of the full-length IN (His-WT) to the SIP1 protein. About 5 µM of the IN60–80 peptide reduced His-WT binding to SIP1 to non-specific background levels, whereas at least 50 µM of the IN231–251 peptide was required to produce a similar effect (Figure 5C). Thus, the inhibitory effect of IN60–80 was more than 10 times higher than that of IN231–251. Of note, the IN60–80 peptide and the IN232–251 peptide significantly reduced the IN-SIP1 mediated RT activity in a dose-dependent manner, while the inhibitory effect was not evident with the control IN154–174 peptide (Figure 5D). The inhibitory effect of IN60–80 (IC_50_ = ∼10 pmoles per reaction) was more than 100 times higher than that of IN231–251 (IC_50_ = ∼1 nmoles per reaction). Interestingly, we also found that synergic inhibitory effect of the IN231–251 with IN60–80 (Figure S5). Thus, although the inhibitory effect of the IN231–251 was much lower than that by the IN60–80 but significant when compared with the control peptide, suggesting contribution of the regions corresponding to the IN231–251, in addition to IN60–80, for efficient binding with SIP1.

Finally, the effect of each peptide on HIV-1 replication was also examined. For this experiment, we used monocyte cell line, THP-1 that was treated with PMA to differentiate to macrophage-like cells to facilitate uptake of the peptide through phagocytosis. Compared to the control peptide IN154–174 or the DMSO control, the IN60–80 peptide significantly reduced HIV-1 infectivity to 20–25% of control (Figure 5E). IN231–251 did not significantly reduce HIV-1 infectivity under these conditions. Furthermore, peptide IN60–80 reduced viral cDNA synthesis to less than 25% of control (Figure 5F). Thus, these data demonstrate that the interaction of HIV-1 IN with SIP1 is critical for efficient reverse transcription of viral RNA after HIV-1 infection. We tested the effect of this peptide on Moloney murine leukemia virus (MMLV) using MMLV-based retroviral vector system (pFB-Luc retroviral vector, Stratagene). MMLV cannot establish in non-dividing cells such as THP-1 cells treated with PMA in which effect of the peptide was evident for HIV-1 cDNA synthesis. Nonetheless, we found that cDNA synthesis was efficiently occurred in these PMA-treated THP-1 cells after infection of MMLV-based VSVG pseudotype vector (Figure S6). Therefore, we examined for effect of the peptides on MMLV cDNA synthesis after infection. We found no significant inhibitory effect of the IN60–80 peptide on MLV cDNA synthesis compared with DMSO or control peptide, ruling out the non-specific inhibitory effects of the IN60–80 during virus binding and entry into the cells.

## Discussion

Contribution of IN during the reverse transcription has been reported not only in HIV-1 [10]–[12], [14], [18], [36] but also in a retrovirus like element of *Saccharomyces cerevisiae*, Ty3 [20], [21]. Physical interaction of HIV-1 IN and RT has been noticed [15], [36], [40], [41] and certain mutations of IN result in viruses defective in early steps of reverse transcription [36], [40]. Furthermore, analysis using cell-free RT assay revealed that IN enhances both initiation and elongation modes of reverse transcription in vitro [19]. These studies suggest that the interaction between IN and RT is functional and critical for viral replication. In this study, we firstly showed that the IN mutations Y15A [28], K186Q, Delta KRK [14], and LL241,242AA [18] that cause reverse transcription defective phenotype, significantly reduced the intracellular interaction with SIP1 (Figure 1). Similar effect was also reproduced in other IN mutations [11] that cause reverse transcription defective phenotype such as C43L and P29F (data not shown). Meanwhile, several other IN variants have been described in the literature to be defective for viral reverse transcription. Some of these IN mutations such as C130S [36], H12A, H16A and F185A [15] reduced the association with RT or RTC. It would be interesting to examine whether these other reverse transcription-defective IN variants also exhibit poor binding to SIP1. These results suggest that the interaction of IN with SIP1 is critical for efficient reverse transcription in the HIV-1 infection cycle. Of note, we confirmed the enhancement of RT activity by IN using in vitro RT assay and the addition of SIP1 in the RT reaction mixture further enhanced RT in the presence of IN but not without IN (Figure 4–5). In this RT assay, we used 3.5∼35 fmoles of RT (corresponding to 0.01∼0.1 units of conventional RT activity) with 0.4∼0.04 fmoles of RNA templates, since if we used higher amount of RT and the template RNA, effect of IN became less evident. The key factor for the IN-mediated RT stimulatory effect is the ratio of amounts of RT and template RNA. In our in vitro RT assay, we used the ratio of RT: RNA 1,000∶1 to 100∶1. Since each mature virus particle contains about 250 molecules of RT [42], our experimental condition might be physiologically relevant to the natural condition during HIV-1 infection cycle. Importantly, the stimulatory effect of SIP1 on RT could not observed when high amount of IN protein was present (Figure S4B). We also noticed the following points; First, SIP1 cannot bind to RT directly. Second, IN can bind to RT directly without SIP1. Third, RT-IN-SIP1 complex can be formed without RNA (data not shown). These data showed that SIP1 could play a critical function as an IN-cofactor, but may not directly interact with the RT. Furthermore, we also found that that pre-incubation of RT and IN before SIP1 resulted in most effective stimulatory effect (Figure S4D). Taken together, these results suggest that IN binds to RT first then with SIP1 to stabilize RT/IN/RNA complex during HIV-1 infection cycle.

We also demonstrate that SIP1 stabilizes the formation of a functional multimer of IN. In cell lines knocked down for LEDGF/p75, which is important for nuclear/chromosomal targeting of HIV-1 IN [7], [43], steady state levels of HIV-1 IN expression were markedly reduced [44]. Interestingly, IN-V5 protein with the Y15A or K186Q mutations were localized predominantly in cytoplasm (data not shown) and exhibited a low level of expression compared with WT-IN (Figure S1A). Therefore, we hypothesize that SIP1 might exert its effect in the cytoplasm, whereas LEDGF/p75 exerts effects in the nucleus to stabilize functional IN conformations that are required for efficient cDNA synthesis and establishing proviral DNA, respectively. It would be of great interest to delineate this point.

The crystal structure of the N-terminal and catalytic core domains (IN1–212) of HIV-1 integrase has been determined [33]. It should be noted that, in the crystal structure of HIV-1 IN1–212, two dimers form a tetramer. In the crystal structure of the IN1–212 tetramer, Y15 is located at the dimer-dimer interface of the integrase tetramer and the side chain of Y15 stacks on the side chain of K186 of other subunit. It is notable that a mutation of K186 was also found to abrogate the infectivity [14]. NMR analysis of an isolated N-terminal domain (IN1–55) has shown that IN1–55 exists in two conformational states, E and D forms [45]. The two forms differ in the coordination of the zinc ion by two histidine residues. Previously, structural analysis of a Y15A mutant by NMR spectroscopy indicated that the mutant protein folds correctly but takes only the E form [28]. Thus, it was suggested that the interaction between Y15 and K186 is required for the optimal tetramerization of integrase, which is required for efficient interaction with SIP1 with the critical motif of IN 60–80 and IN 231–251, locates in the core and the C-terminal domain, respectively.

Recent study using a large-scale small interfering RNA screen identified more than 250 host factors required for HIV-1 infection [46], [47]. Interaction of these host factors and virus could be possible targets for development of new class of HIV-1 inhibitors. SIP1 was noticed as one of the host factors required for HIV-1 infection in the genome-wide screening analysis [46]. Thus, targeting the IN-SIP1 interaction might provide beneficial therapeutic results. The dimer form of SIP1 interacts with dimer forms of SMN and enhances the SMN oligomer formation and the assembly activity of snRNP [48]. It is unclear which form of IN could interact with dimer form of SIP1. In our present study, we showed that IN-Y15A was defective for multimerization and had a poor binding ability with SIP1, and low stability in vivo. In vitro, SIP1 are capable of interacting with recombinant IN-Y15A in which formed the dimer, but not tetramer (data not shown). These results suggest that similar to interaction with SMN, SIP1 may recognize the dimer form of IN preferentially and play an important role of the stabilization of functional high-order structure forms of IN. In addition, IN-derived peptide (IN60–80) corresponding to one of the IN motif essential for binding to SIP1 specifically block IN-mediated RT stimulatory effect and HIV-1 replication. The IN60–80 peptide does not directly suppress viral entry and RT enzymatic activity (data not shown). These results suggest the critical role of the IN-SIP1 interaction in reverse transcription in viral replication cycle. Since SIP1 has critical cellular functions, it is obvious that drugs should be highly specific for the IN-SIP1 interface.

In summary, we demonstrated here that HIV-1 IN and SIP1 synergistically augment viral cDNA synthesis by enhancing the assembly of RT on viral RNA *in vitro*. In addition, the disruption of the SIP1-IN interaction with synthetic peptides confirmed the critical role of the IN and SIP1 interaction in reverse transcription and the HIV-1 replication cycle. These results could serve as a basis for novel approaches in the development of HIV-1 inhibitors that target IN and host factor interactions [49], [50].

## Materials and Methods

### Plasmids

To create plenti-IN-V5, pNL4-3 [51] was amplified with the sense primer and antisense primer. The resulting fragments were cloned into pENTR using the TOPO Cloning Kits (Invitrogen), and transferred into pLenti6/V5-DEST (Invitrogen) by LR recombination. The human beta-globin intron sequences were introduced in pLenti6/V5-DEST using the *Nde*I and *Eco*RI sites. To generate the GST-tagged SIP1 bacterial expression construct pGEX-SIP1, the SIP1 gene was amplified from pTRE-SIP1 (kindly provided by Dr. G. Dreyfuss) by PCR and cloned into *Bam*HI and *Eco*RI sites of pGEX-6P-2 (Amersham Pharmacia Biotech). To generate pQE30-IN for the bacterial expression of His-tagged HIV-1 IN, the IN gene was obtained by PCR of pNL4-3 and was inserted into pQE30 (QIAGEN) between *Bam*HI and *Sal*I sites.

### Immunoprecipitation

The plasmids encoding integrase or its mutants were transfected into 293T cells using lipofectamine 2000 (Invitrogen). At 48 h post-transfection, transfected cells were harvested and suspended in 0.8 ml RSB-100 (10 mM Tris-HCl, pH 7.5, 100 mM NaCl, and 2.5 mM MgCl_2_) with 1% Nonidet P-40. Cell lysates were centrifuged at 15,000×*g* for 20 min at 4°C. The supernatant was incubated with 4 µg of anti-V5 polyclonal antibody (Delta Biolabs) and 40 µl of protein G-Sepharose 4B Fast Flow beads (Amersham Pharmacia Biotech) for 2 h at 4°C. The beads were washed with wash buffer A (20 mM Tris-HCl, pH 7.5, 250 mM NaCl, 1 mM EDTA, 0.5 M LiCl 5% glycerol, 1% TritonX-100, and 0.25% Nonidet P-40) and then with RSB-100 containing 1% Nonidet P-40. The immunocomplex was eluted by boiling with 20 µl of 5× sample buffer and analyzed by SDS-PAGE and Western blot.

### *In Vitro* Binding Assay

10 µg of His-tagged integrase or its deletion mutant was incubated with 10 µg of SIP1 and 30 µl of Ni-NTA agarose (QIAGEN) in storage buffer (20 mM HEPES pH 7.5, 0.1 mM EDTA, 300 mM NaCl, 10 mM chaps, and 20% glycerol) at 4°C. After a 2 h incubation, beads were washed with wash buffer A and RSB-100 containing 1% Nonidet P-40, then boiled in 20 µl of 5× sample buffer. The samples were subjected to SDS-PAGE followed by Western blot analysis.

### Dot-Blot Binding Assay

His-tagged integrase (10 µg) or IN-derived peptides were bound to the nitrocellulose membranes for 30 min. The blots were incubated with blocking buffer for 16 h at 4°C. After washing with TBS-T (20 mM Tris-HCl pH 7.4, 135 mM NaCl, and 0.05% Tween 20), the membranes were incubated with 10 µg of SIP1 in blocking buffer for 2 h at room temperature, followed by washing with TBS-T. The blots were incubated with mouse anti-SIP1 antibodies for 2 h at room temperature, and then washed with TBS-T. After the incubation was performed with horseradish peroxidase-conjugate anti-mouse IgG antibodies for 1 h at room temperature, blots were washed and SIP1 was detected using ECL solution.

### Cross-Linking

293T cells were lysed with CSK buffer (10 mM Pipes pH 6.8, 10% (w/v) sucrose, 1 mM dithiothreitol, 1 mM MgCl_2_, 400 mM NaCl, and 0.5% Nonidet P-40) [5]. Then, cell lysates were incubated with 3,3′-Dithiobis [sulfosuccinimidylpropionate] (DTSSP) at room temperature for 20 min. The cross-linking reaction was terminated by the addition of 5× sample buffer.

### Transfection of siRNA

Control (5′-CAA GGA CGU UCU AAG GUG GAG AGC U-3′) and SIP1 (5′-CCU UGC UUA GUA UUG UUA GCA GAA U-3′)-specific siRNAs were purchased from Invitrogen. 293T cells were transfected with 40 nM of siRNA using Lipofectamine 2000 (Invitrogen).

### Purification of Recombinant IN and SIP1

The plasmids pGEX-SIP1, pQE30-IN encoding GST-tagged SIP1, or His-tagged IN were transformed into *E. coli* BL21 (pGEX-SIP1) or M15 (pQE30-IN). To purify GST-SIP1, cells were cultured in LB medium at 37°C for 16 h, diluted at 1∶40 in fresh LB medium containing 100 µg/ml ampicillin and incubated at 37°C. When the cells reached a density of OD_600_ = 0.8, IPTG was added to reach a final concentration of 1 mM. The cells were harvested by centrifuging at 5000×g, and lysed with PBS containing 1 M NaCl, 1% TritonX-100, and 3 mM DTT. The cell lysates were sonicated and centrifuged for 1 h at 24,000×g. The supernatant was incubated in a glutathione Sepharose 4B column (Amersham Pharmacia Biotech). The columns were washed with phosphate-buffered saline (PBS) containing 1 M NaCl and 1% TritonX-100. GST-tagged SIP1 was eluted with 2 ml of elution buffer (15 mM reduced glutathione, 1 mM EDTA, 150 mM NaCl, and 20 mM Tris-HCl, pH 7.5). Purified GST-tagged SIP1 was cleaved from GST by PreScission protease (Amersham Pharmacia Biotech). To prepare His-IN, lysis of bacteria was carried out using the same procedure as described for GST-SIP1. His-tagged IN was bound to Ni-NTA agarose, washed with PBS containing 50 mM imidazole, 1 M NaCl, 1% TritonX-100 and 3 mM DTT, and eluted with 500 mM imidazole containing 1 mM EDTA, 150 mM NaCl, 20 mM Tris-HCl, pH 7.5 and 1% TritonX-100. Proteins were then dialyzed against storage buffer (300 mM NaCl, 20 mM HEPES, pH 7.5, 1 mM EDTA, 1 mM DTT, and 20% (w/v) glycerol).

### *In Vitro* RT Assay

For preparation of the viral RNA template for *in vitro* transcription, the DNA sequences corresponding to HIV-1 lentiviral genomic RNA (pCS-CDF-CG-PRE) [52] were subcloned into pGEM-T vector (pGEM-CS-CDF). Then, the annealed oligonucleotides of TCGA(A)_30_G and TCGA(T)_30_C were inserted into the pGEM-CS-CDF vector at the Sal-I site for addition of the poly(A) tail (the pGEM-CS-CDF-PolyA). Synthesized HIV RNA with the poly(A)_ 30_ tail were produced by *in vitro* transcription using RiboMAX™ Large Scale RNA Production System-T7 (Promega) with Ribo m^7^ G Cap analog (Promega) to mimic the capped structure of mRNA. Transcription products of HIV-1 RNA (4.9 kb) were purified with spin columns MicroSpin™ G-25 Columns (American Biosciences), followed by gel-filtration using NAP-5 columns, (American Biosciences) and were used in the *in vitro* RT assay. The reverse transcription reactions were carried out in a final volume of 20 µl RT reaction buffer consisting of 50 mM Tris-HCl (pH 8.3), 75 mM KCl, 3 mM MgCl_2_, 10 mM DTT, 1 mM dNTPs, 0.04∼0.4 fmoles of HIV-1 RNA templates and 0.4∼4 fmoles of RNA primer, 3.5∼35 fmol HIV-1 RT (GST-RTp66). The reaction was carried out at 42°C for 30 min in either the absence or presence of different amount of His-IN and recombinant SIP1. The amount of the cDNA product was measured by real-time PCR using primers for the R/U5 or R/gag region [11].

## Supporting Information

Figure S1 IN mutants showing lowered affinity with SIP1 are unstable in 293T cells. (A) 293T cells were transfected with 1 µg of V5-tagged IN (WT) or its mutants or LacZ expression plasmid. At 48 after transfection, cells were suspended with 400mCSK buffer containing 0.5% NP-40. Cell lysates were separated on SDS-PAGE gel and analyzed by western blotting using anti-V5 antibody. (B) V5-tagged IN containing Y15A alone or Delta-KRK and Y15A mutations was analyzed as described in (A).
(14.18 MB TIF)

Figure S2 Recombinant IN mutants showing RT defective phenotype interact with recombinant SIP1 *in vitro*. The recombinant SIP1 (10 µg) was incubated with His-tagged IN (10 µg) coupled to nickel agarose. The complexes were precipitated and performed western blotting using anti-SIP1 antibody. NC indicates that 10 µg of recombinant SIP1 was incubated with nickel agarose in the absence of His-tagged IN.
(6.01 MB TIF)

Figure S3 The subcellular localization of IN does not alter in SIP1-deficient cells. At 48 h after transfection of siRNA and IN-V5 expression plasmid, 293T cells were fractionated into cytoplasmic, membrane, nuclear and cytoskeleton. Each fraction was analyzed by western blotting using anti-V5 antibody, anti-SIP1 antibody, or anti-histone H3 antibody as nuclear fraction control.
(6.01 MB TIF)

Figure S4 Stoichiometry and order of IN and SIP1 for their stimulatory effect of cDNA synthesis. (A) The *in vitro* RT assay was carried out in the absence or presence of different amount of the His-IN (0.2 or 1 pmol) or recombinant SIP1 (0.2 or 1 pmol) as described in Figure 4. The amount of cDNA product was measured by real-time PCR using primers for HIV-1 R/U5 region. (B) The *in vitro* RT assay was carried out with 5 pmoles of His-IN in the absence or presence of different amount SIP1 (1 or 5 pmol). (C) The *in vitro* RT assay was carried out in the absence or presence of the His-IN and recombinant SIP1 with [α32P]dCTP and subjected to SDS-PAGE analysis in denatured condition. (D) *In vitro* RT assay was performed as described above, except that rIN with rRT and rSIP1 were pre-incubated on ice in different combinations and orders (inlet Table). Then, reaction was initiated by adding mixture containing the template RNA/PBS-primer and dNTPs.
(0.10 MB PDF)

Figure S5 Synergistic inhibitory effect of IN60–80 and IN231–251. The *in vitro* reverse transcription assay was performed with 35 fmol of RT, 3.5 fmol of His-IN, and 50 fmol of rSIP1 in either the absence (DMSO control) or presence of 1 nmole of each IN-derived peptide or combinations containing 0.5 nmoles of each peptide.
(0.04 MB PDF)

Figure S6 Effect of the IN-derived peptide on MLV cDNA synthesis. PMA-stimulated THP-1 cells were treated with 100 µM of IN-derived peptide for 16 h. Cells were infected with Moloney murine leukemia virus (MMLV)-based retroviral vector (pFB-Luc retroviral vector, Stratagene) in the presence of 100 µM of IN-derived peptide for 6 h. At 24 h post-infection, the level of MLV cDNA synthesis for early (R/U5) products of reverse transcription in cells.
(0.04 MB PDF)
