# Supplementary figures and images for: Augmentation of Reverse Transcription by Integrase through an Interaction with Host Factor, SIP1/Gemin2 Is Critical for HIV-1 Infection
Source: PLoS One. 2009 Nov 13;4(11):e7825. doi: 10.1371/journal.pone.0007825 (PMC2771899; doi:10.1371/journal.pone.0007825)

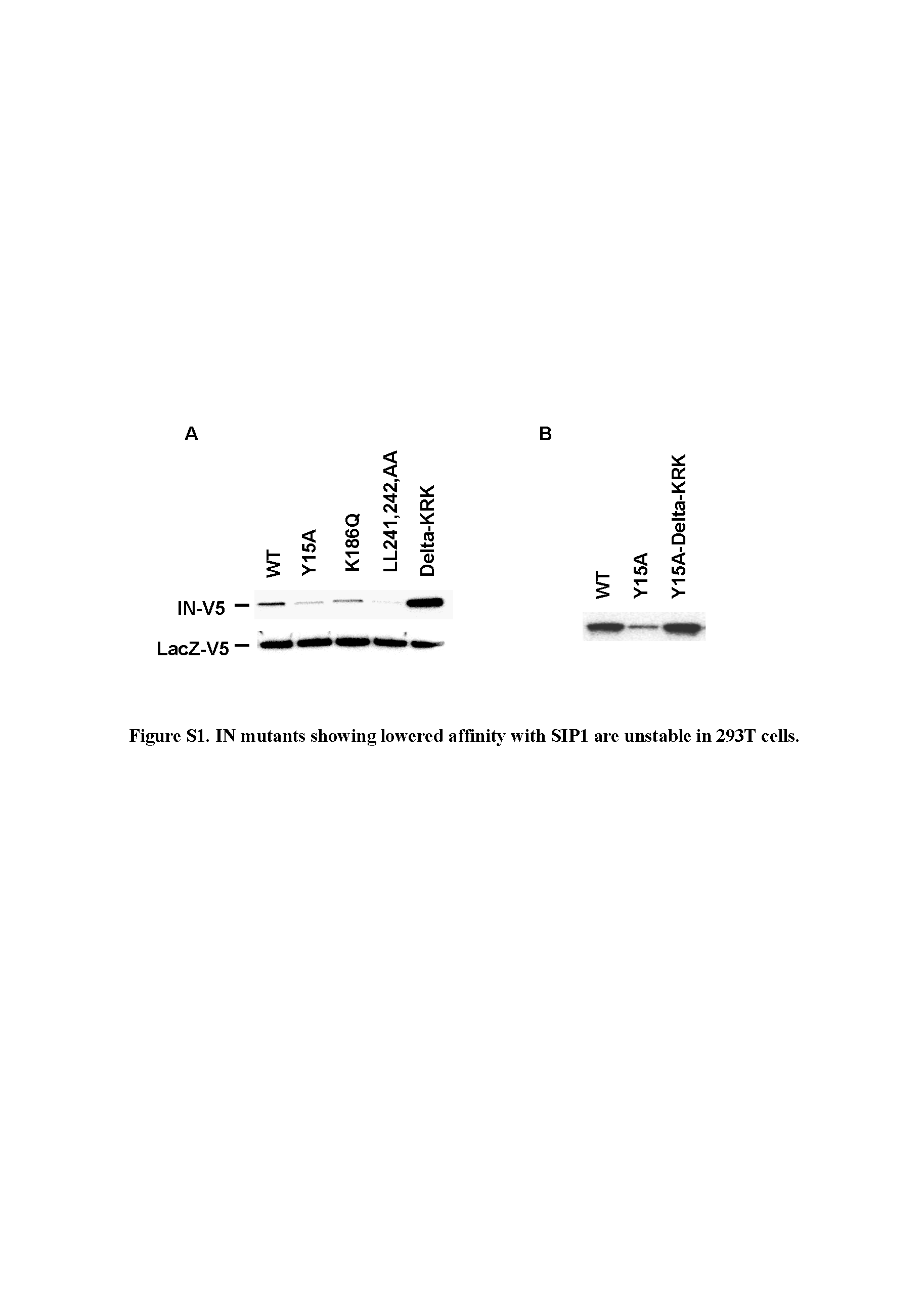

Supplement: Figure S1 — IN mutants showing lowered affinity with SIP1 are unstable in 293T cells. (A) 293T cells were transfected with 1 µg of V5-tagged IN (WT) or its mutants or LacZ expression plasmid. At 48 after transfection, cells were suspended with 400mCSK buffer containing 0.5% NP-40. Cell lysates were separated on SDS-PAGE gel and analyzed by western blotting using anti-V5 antibody. (B) V5-tagged IN containing Y15A alone or Delta-KRK and Y15A mutations was analyzed as described in (A). (14.18 MB TIF) [file pone.0007825.s001.tif]

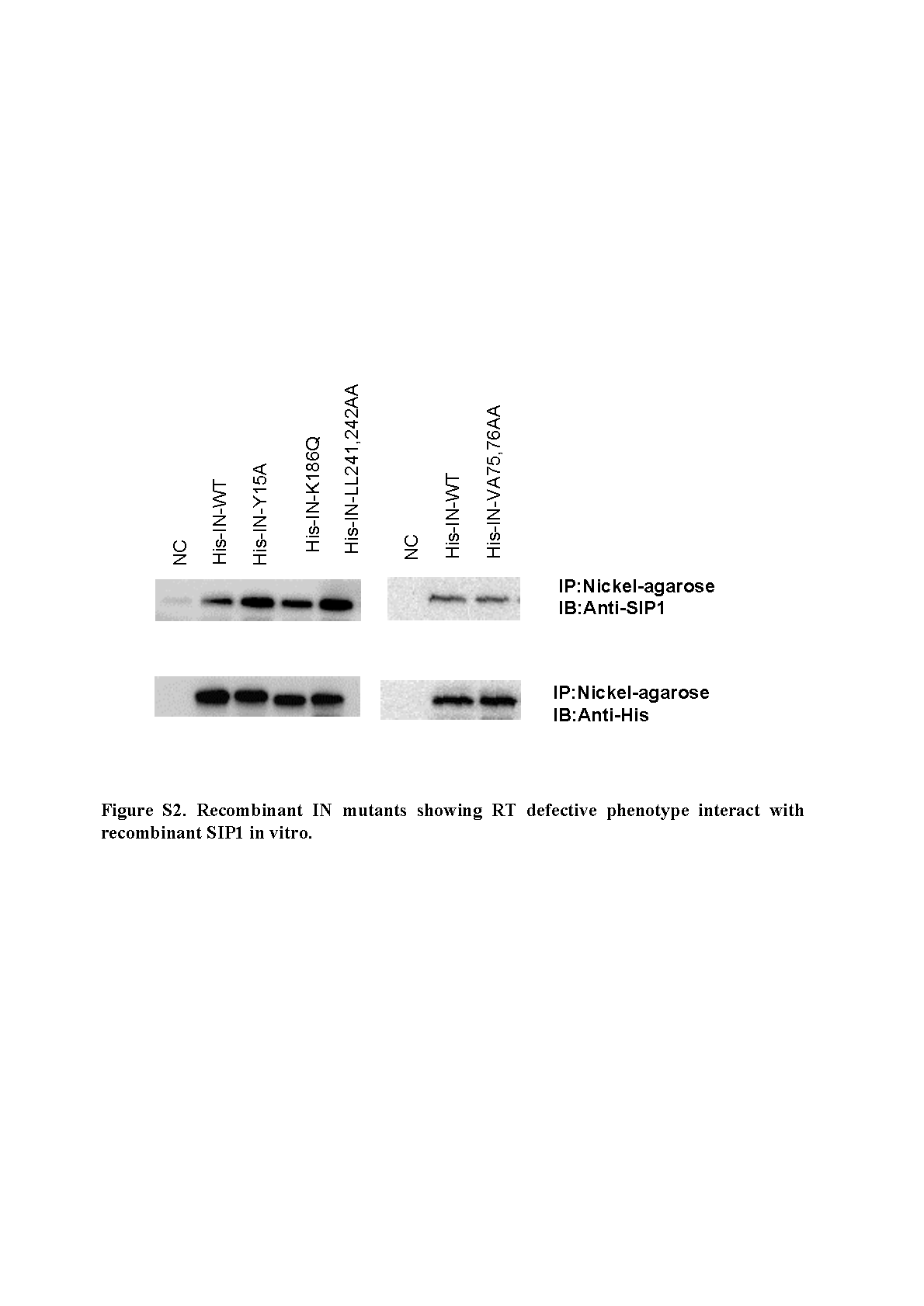

Supplement: Figure S2 — Recombinant IN mutants showing RT defective phenotype interact with recombinant SIP1 in vitro. The recombinant SIP1 (10 µg) was incubated with His-tagged IN (10 µg) coupled to nickel agarose. The complexes were precipitated and performed western blotting using anti-SIP1 antibody. NC indicates that 10 µg of recombinant SIP1 was incubated with nickel agarose in the absence of His-tagged IN. (6.01 MB TIF) [file pone.0007825.s002.tif]

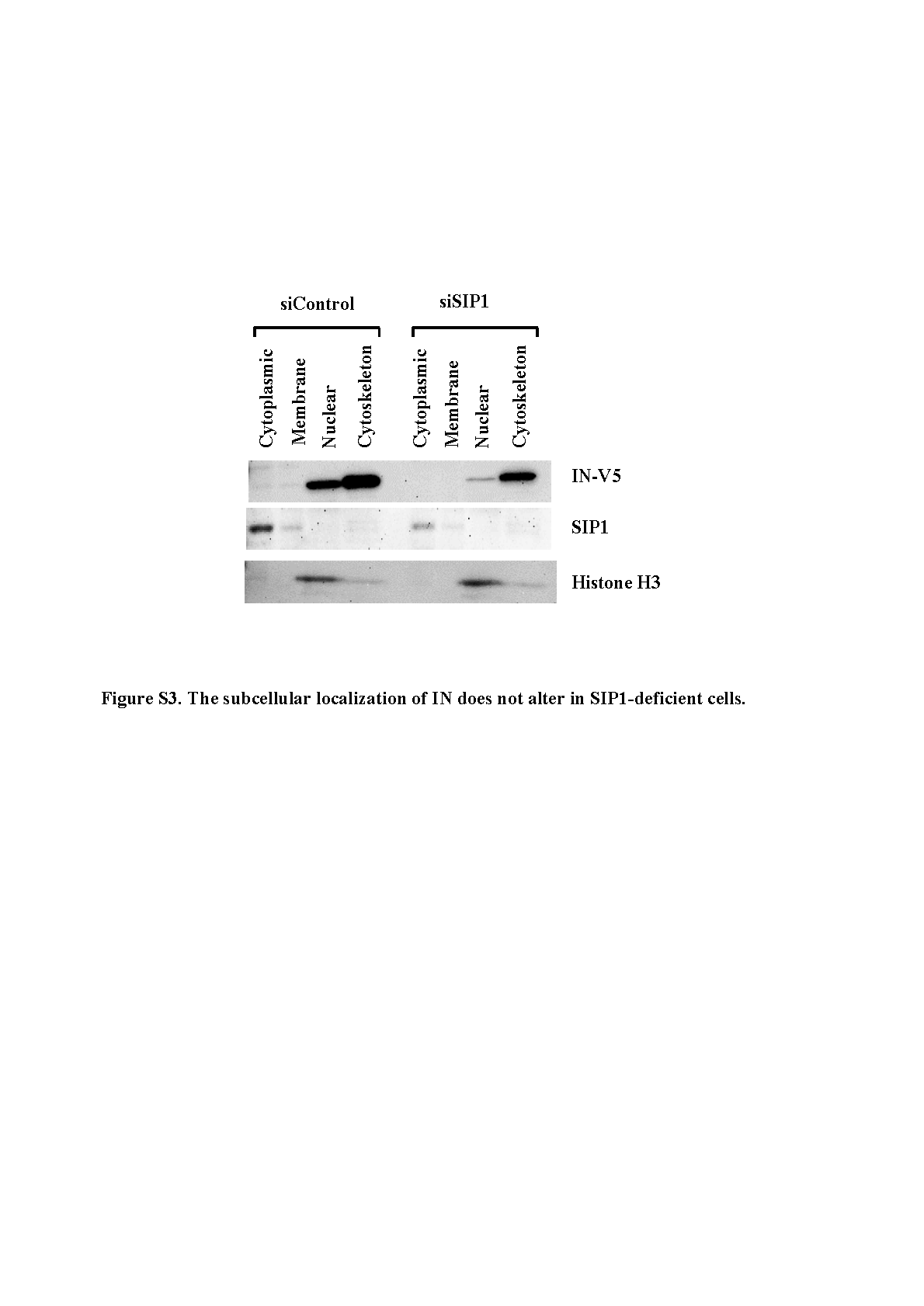

Supplement: Figure S3 — The subcellular localization of IN does not alter in SIP1-deficient cells. At 48 h after transfection of siRNA and IN-V5 expression plasmid, 293T cells were fractionated into cytoplasmic, membrane, nuclear and cytoskeleton. Each fraction was analyzed by western blotting using anti-V5 antibody, anti-SIP1 antibody, or anti-histone H3 antibody as nuclear fraction control. (6.01 MB TIF) [file pone.0007825.s003.tif]

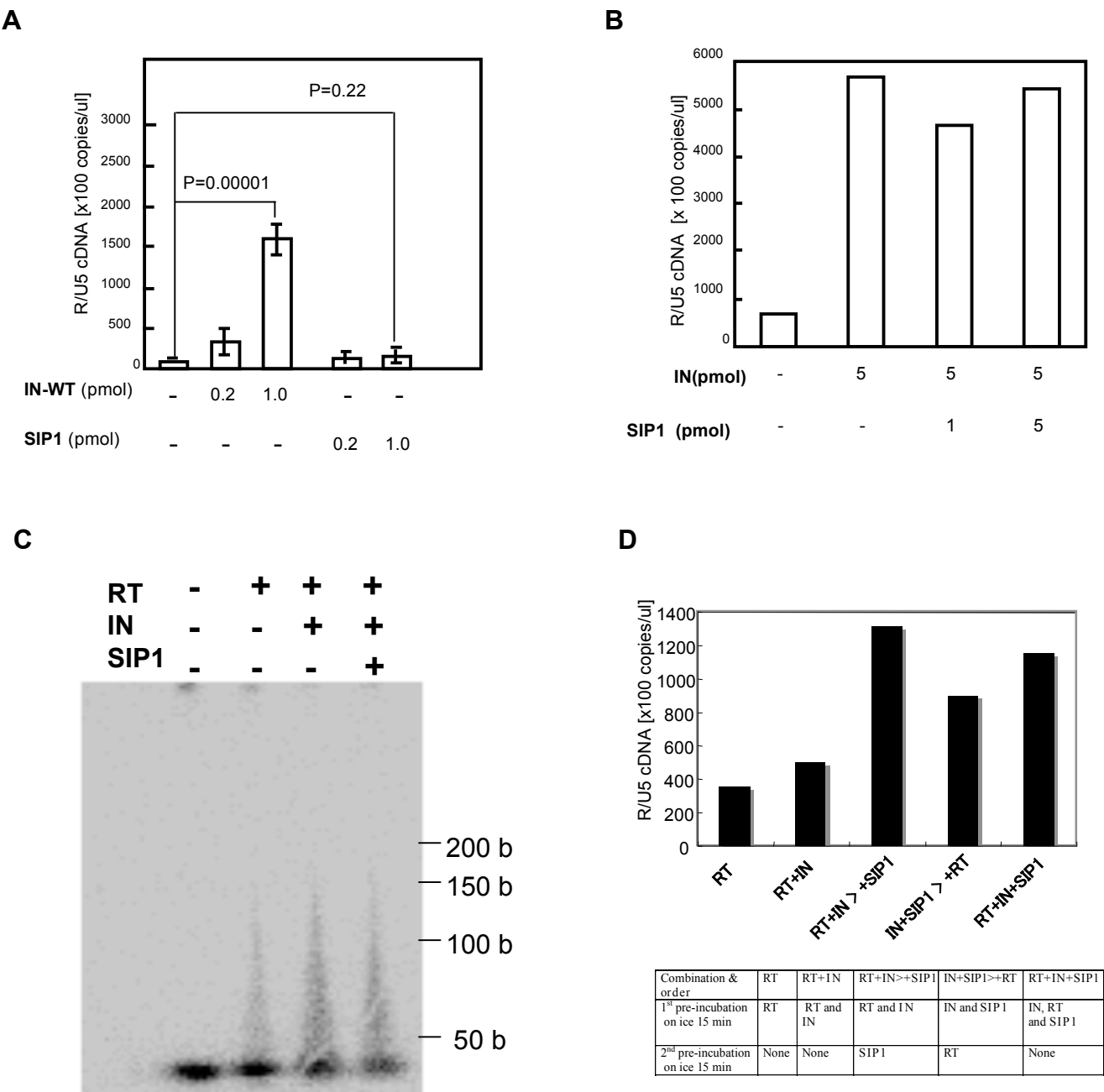

**Figure S4. Stoichiometry and order of IN and SIP1 for their stimulatory effect of cDNA synthesis.**

Supplement: Figure S4 — Stoichiometry and order of IN and SIP1 for their stimulatory effect of cDNA synthesis. (A) The in vitro RT assay was carried out in the absence or presence of different amount of the His-IN (0.2 or 1 pmol) or recombinant SIP1 (0.2 or 1 pmol) as described in Figure 4. The amount of cDNA product was measured by real-time PCR using primers for HIV-1 R/U5 region. (B) The in vitro RT assay was carried out with 5 pmoles of His-IN in the absence or presence of different amount SIP1 (1 or 5 pmol). (C) The in vitro RT assay was carried out in the absence or presence of the His-IN and recombinant SIP1 with [α32P]dCTP and subjected to SDS-PAGE analysis in denatured condition. (D) In vitro RT assay was performed as described above, except that rIN with rRT and rSIP1 were pre-incubated on ice in different combinations and orders (inlet Table). Then, reaction was initiated by adding mixture containing the template RNA/PBS-primer and dNTPs. (0.10 MB PDF) [file pone.0007825.s004.pdf]

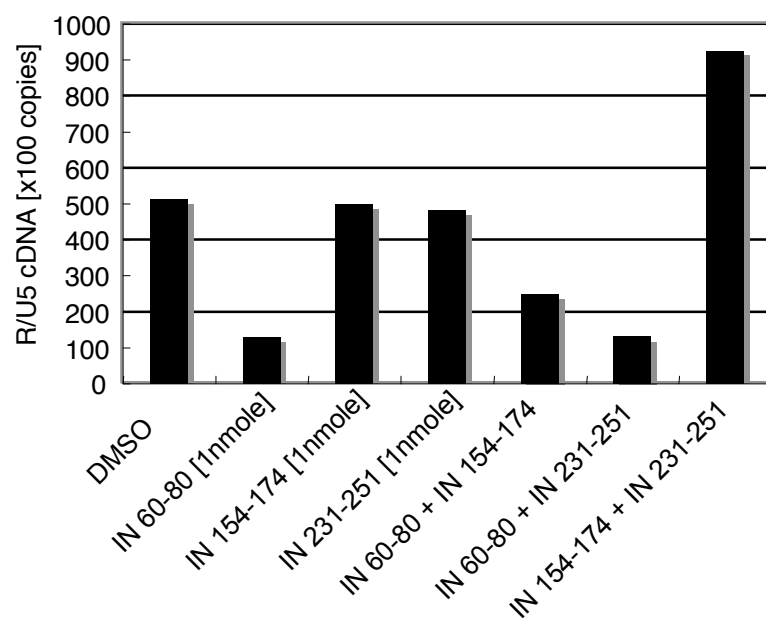

**Figure S5. Synergistic inhibitory effect of IN60-80 and IN231-251.**

Supplement: Figure S5 — Synergistic inhibitory effect of IN60–80 and IN231–251. The in vitro reverse transcription assay was performed with 35 fmol of RT, 3.5 fmol of His-IN, and 50 fmol of rSIP1 in either the absence (DMSO control) or presence of 1 nmole of each IN-derived peptide or combinations containing 0.5 nmoles of each peptide. (0.04 MB PDF) [file pone.0007825.s005.pdf]

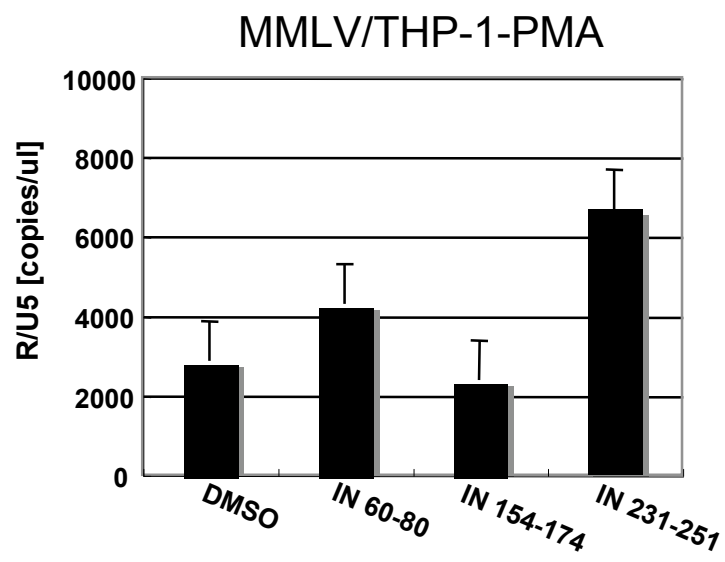

**Figure S6. Effect of the IN-derived peptide on MLV cDNA synthesis.**

Supplement: Figure S6 — Effect of the IN-derived peptide on MLV cDNA synthesis. PMA-stimulated THP-1 cells were treated with 100 µM of IN-derived peptide for 16 h. Cells were infected with Moloney murine leukemia virus (MMLV)-based retroviral vector (pFB-Luc retroviral vector, Stratagene) in the presence of 100 µM of IN-derived peptide for 6 h. At 24 h post-infection, the level of MLV cDNA synthesis for early (R/U5) products of reverse transcription in cells. (0.04 MB PDF) [file pone.0007825.s006.pdf]
